# Copper Oxide Nanoparticles Induce Autophagic Cell Death in A549 Cells

**DOI:** 10.1371/journal.pone.0043442

**Published:** 2012-08-20

**Authors:** Tingting Sun, Yiwu Yan, Yan Zhao, Feng Guo, Chengyu Jiang

**Affiliations:** State Key Laboratory of Medical Molecular Biology, Institute of Basic Medical Sciences, Chinese Academy of Medical Sciences, Peking Union Medical College, Tsinghua University, Beijing, China; IISER-TVM, India

## Abstract

Metal oxide nanoparticles (NPs) are among the most highly produced nanomaterials, and have many diverse functions in catalysis, environmental remediation, as sensors, and in the production of personal care products. In this study, the toxicity of several widely used metal oxide NPs such as copper oxide, silica, titanium oxide and ferric oxide NPs, were evaluated *In vitro.* We exposed A549, H1650 and CNE-2Z cell lines to metal oxide NPs, and found CuO NPs to be the most toxic, SiO2 mild toxic, while the other metal oxide NPs had little effect on cell viability. Furthermore, the autophagic biomarker LC3-II significantly increased in A549 cells treated with CuO NPs, and the use of the autophagy inhibitors wortmannin and 3-methyladenin significantly improved cell survival. These results indicate that the cytoxicity of CuO NPs may involve the autophagic pathway in A549 cells.

## Introduction

Metal oxide nanoparticles have been used to produce a wide range of products. Their industrial and commercial applications include catalysis, sensors, environmental remediation, personal care products, cosmetics, and they are showing great prospect in the field of medicine, including imaging and drug delivery [1]–[3]. The massive increase in the manufacturing and utilization of nanoparticles, however, has generated major concerns regarding the harmful effects these particles may have on human health and the environment [4]–[6]. Several studies focusing on metal oxide NPs have demonstrated that these NPs have toxic effects in cells and organisms. For example, it has been reported that metal oxide nanoparticles cause genotoxicity, mitochondrial dysfunction and increased cell death in some cell lines [7]–[9]. ZnO and CuO NPs have been shown to have toxic effects in bacteria, yeast, microalgae, crustaceans, and zebrafish [10]–[13]. Additional studies, however, are needed to further evaluate the toxicity of these nanoparticles and to determine their potential threat to human health.

Nanoparticles have a small aerodynamic diameter. They can easily penetrate lung tissue and cause adverse pulmonary reactions [14]. In this study we used respiratory epithelial cell lines to compare the cytotoxicity of a panel of metal oxide NPs, which included CuO, SiO_2_, TiO_2_, Fe_2_O_3_ and Fe_3_O_4_. Furthermore, the mechanism of cell death caused by metal oxides NPs was investigated.

## Materials and Methods

### Nanoparticles and Reagents

Copper (II) oxide (<50 nm), iron (III) oxide (<50 nm), iron (II, III) oxide (<50 nm), and silicon dioxide (5–15 nm) nanopowder were purchased from Sigma Aldrich; titanium (IV) oxide anatasenanopowders (10 nm and 32 nm) were purchased from Alfa Aesar. 3-Methyladenine, wortmannin, zVAD-fmk, necrostatin 1, rapamycin and bafilomycin A1 were purchased from Sigma. siRNAs against human Atg5 (5′-ACCGGAAACUCAΜGGAAUAdTdT-3′/3′-dTdTΜGGCCUUΜGAGUACCUUAU-5′) were purchased from RiBo Biotechnology. Transfection reagent X-treme Gene HP was purchased from Roche and Lipo2000 was purchased from Invitrogen.

LC3B and cleaved caspase-3 primary antibodies were purchased from Cell Signaling Technologies. Anti-β-actin antibody was purchased from Sigma-Aldrich. Horseradish peroxidase-conjugated secondary antibodies and western blot luminal reagents were purchased from Santa Cruz Biotechnology. The Celltiter 96 Aqueous One Solution Cell Proliferation Assay kit was obtained from Promega. The in situ cell death detection kit-POD was purchased from Roche.

The EGFP-LC3 plasmid, which encodes a fusion protein of enhanced green fluorescent protein (EGFP) and LC3, was constructed by K.Kirkegaard (Department of Microbiology and Immunology, Stanford University School of Medicine, Stanford, California) and was obtained from ADDGENE.

### Cell Culture and Exposure to Nanoparticles and Drugs

Human adenocarcinoma A549 cells, human non-small cell lung cancer H1650 cells and human nasopharyngeal carcinoma CNE-2Z cells were provided by American Type Culture Collection (ATCC). The human type II alveolar epithelial cell line (A549) was cultured in F12/HAM’s (Hyclone) medium supplemented with 10% FBS and 100 U/mlpenicillin-streptomycin at 37°C and 5% CO_2_. NCI-H1650 and CNE-2Z were culturedin RMPI-1640 medium at 37°C and 5% CO_2_. Nanoparticles were suspended in culture medium at a concentration of 1 mg/ml, and then sonicated in a sonicator bath for 30 min. The solution was then diluted with medium to a concentration of 30 µg/ml. The dilutions of NPs were vigorously vortexed for 30 s prior to cell exposure to avoid nanoparticle agglomeration. Bafilomycin was diluted in DMSO at a concentration of 50 µM and then add to the cells at a concentration of 50 nM 1 h before exposure to nanoparticles.

### MTT Assay

The cytotoxic potential of the metal oxides was assessed using the MTS assay. A549, NCI-H1650 and CNE-2Z were seeded at a concentration of 1×10^5^/ml in96-well plates and then exposed to metal oxide NPs 12 h later at a concentration of 30 µg/ml for 24 h, the function of bafilimycin A1 on A549 cells were treated with CuO nanoparticles of 30 µg/ml for 18 h. For each well, the medium was removed and replaced with 100 µl of fresh medium and 20 µl of Celltiter 96 Aqueous One Solution Cell Proliferation Assay solution. The cells were incubated at 37°C for 1 h before the absorbency was measured at 490 nm. In the assays using cell death inhibitors, 3-methyladenin, wortmannin, zVAD-fmk, and necrostatin 1 were diluted to proper concentrations in cell culture medium and added shortly before the cells were exposed to nanoparticles.

### TUNEL Assay

A549 cells were seeded at a concentration of 1×10^5^ cells/ml in 24-well plates and incubated for 12 h. Cells were then exposed to either 30 µg/ml CuO or 30 µg/ml SiO_2_ nanoparticles for 8 h. Samples were examined for apoptosis using the *in situ* cell death detection kit according to the manufacturer’s instruction manual.

### Western Blot Analysis

For LC3 analysis, A549 cells were seeded at a concentration of 1×10^5^ cells/ml in 24-well plates and incubated overnight, cells were treated with 30 µg/ml CuO or 30 µg/ml SiO_2_nanoparticles for 12 h. For autophagy flux analysis, A549 cells were seeded at a concentration of 5×10^5^ cells/ml in 6-well plates and incubated overnight, cells were treated with 50 nM bafilomycin A1 for 1 hour before added 30 µg/ml CuO nanoparticles for 3 h. Cells were lysed with ice-cold lysis buffer [50 mM tris-HCl (pH 7.5),150 mM NaCl, 1.0% Triton X-100, 20 mM EDTA, 1 mM Na_3_VO_4_, 1 mM NaF, and protease inhibitors], then the same volume of 2× Loading Buffer [10% SDS, 5% sucrose, 0.1% bromophenol blue and 5% β-mercaptoethanol] were added, samples were splitted on ice for 15 min and then denatured at 95°C for 15 min. Samples were subjected to Western blot analysis, and band densities were analyzed using AlphaEaseFC or Quantity One software.

### Transmission Electron Microscopy

A549 cells were treated with CuO (30 µg/ml), SiO2 NPs (30 µg/ml) or rapamycin (5 µM) for 12 h. Cells were then collected and fixed with 2.5% glutaraldehyde. Samples were then fixed in 1% OsO4 for 1 h, dehydrated by increasingconcentrations of acetone, and gradually infiltrated with epoxy resin. Ultra-thin sections were stained with uranyl acetate and lead citrate. The percentage of autophagy-positive cells was calculated. A cell showing two or more autophagosomes was defined to be an autophagy-positive cell.*P<0.05; **P<0.01.

### Analysis of LC3-EGFP Aggregates

A549 cells were seeded on coverslips in 12-well plates, and 24 h later, transfected with LC3-EGFP plasmid. 24 h after transfection, cells were incubated with CuO (30 µg/ml), SiO_2_ NPs (30 µg/ml) or rapamycin (5 µM) for 6 h at 37°C and 5% CO_2_. EGFP^+^ dots in the cell were counted via Olympus laser-scanning spectrum confocal system linked to a microscope. Images were captured under the 100× oil objective with the confocal acquisition software FV10-ASW 3.0. A cell containing three or more EGFP^+^ dots was defined as an LC3-positive cell.

### Knockdown of *Atg5* in A549 Cells

A549 cells were seeded in a 24-well plate, and 24 h later, treated with control siRNA or ATG5-specific siRNA duplex (100 µg in 100 µl) in DEPC-treated saline. After 36 h, knockdown efficiency was determined by Western blotting with anti-Atg5 antibody. 36 h after transfection, cells were digested with trypsin and seeded on 96-well plates. 12 h later, CuO NPs (30 µg/ml) was added to the Atg5 siRNA and control siRNA group in the 96-well plate, and the MTS assay was conducted on the next day. In addition, siRNA-treated cells were also subject to Western blotting after CuO exposure for detection of LC3.

### Results Copper Oxide is Toxic

**Figure 1:**
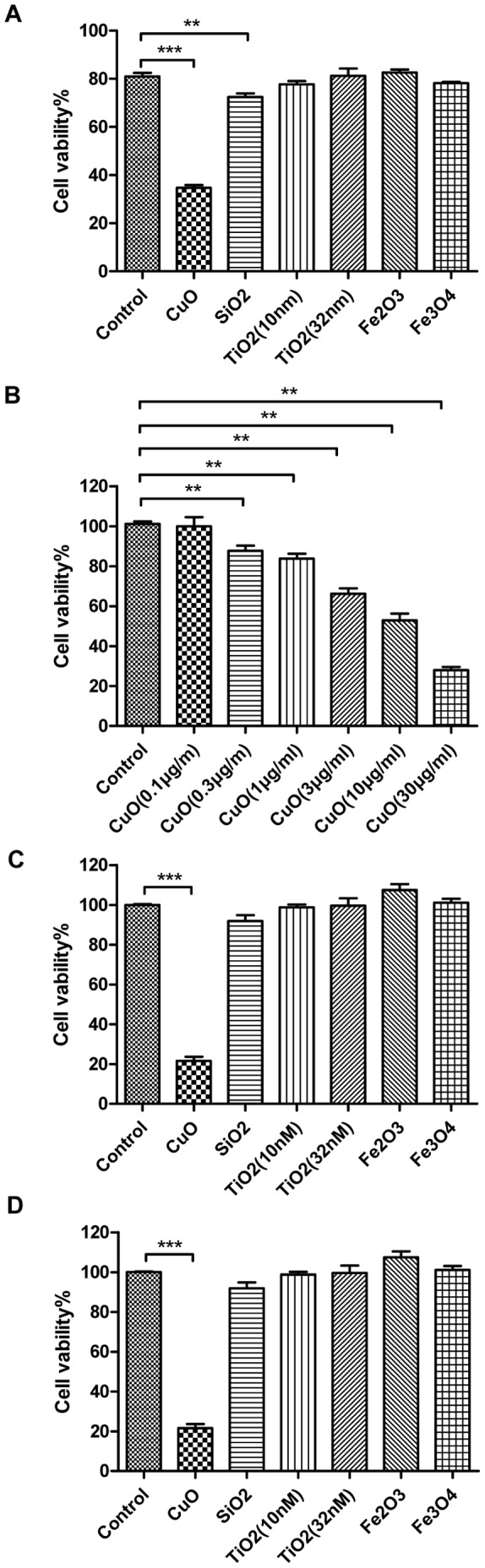
Copper oxide nanoparticles cause cell death in respiratory cell lines. (A) MTS assay results for A549 cells treated with CuO, SiO_2_, TiO_2_ (10 nm, 32 nm), Fe_2_O_3_ or Fe_3_O_4_ NPs (30 µg/ml) for 24 h (B) MTS assay results for A549 cells treated with CuO NPs (0.1, 0.3, 1, 3, 10 and 30 µg/ml) for 24 h (C) MTS assay results for H1650 cells treated with NPs (30 µg/ml) for 24 h (D) MTS assay results for CNE-2Z cells treated with NPs (30 µg/ml) for 24 h. ***p<0.001 versus control and **p<0.01 versus control.

**Figure 2:**
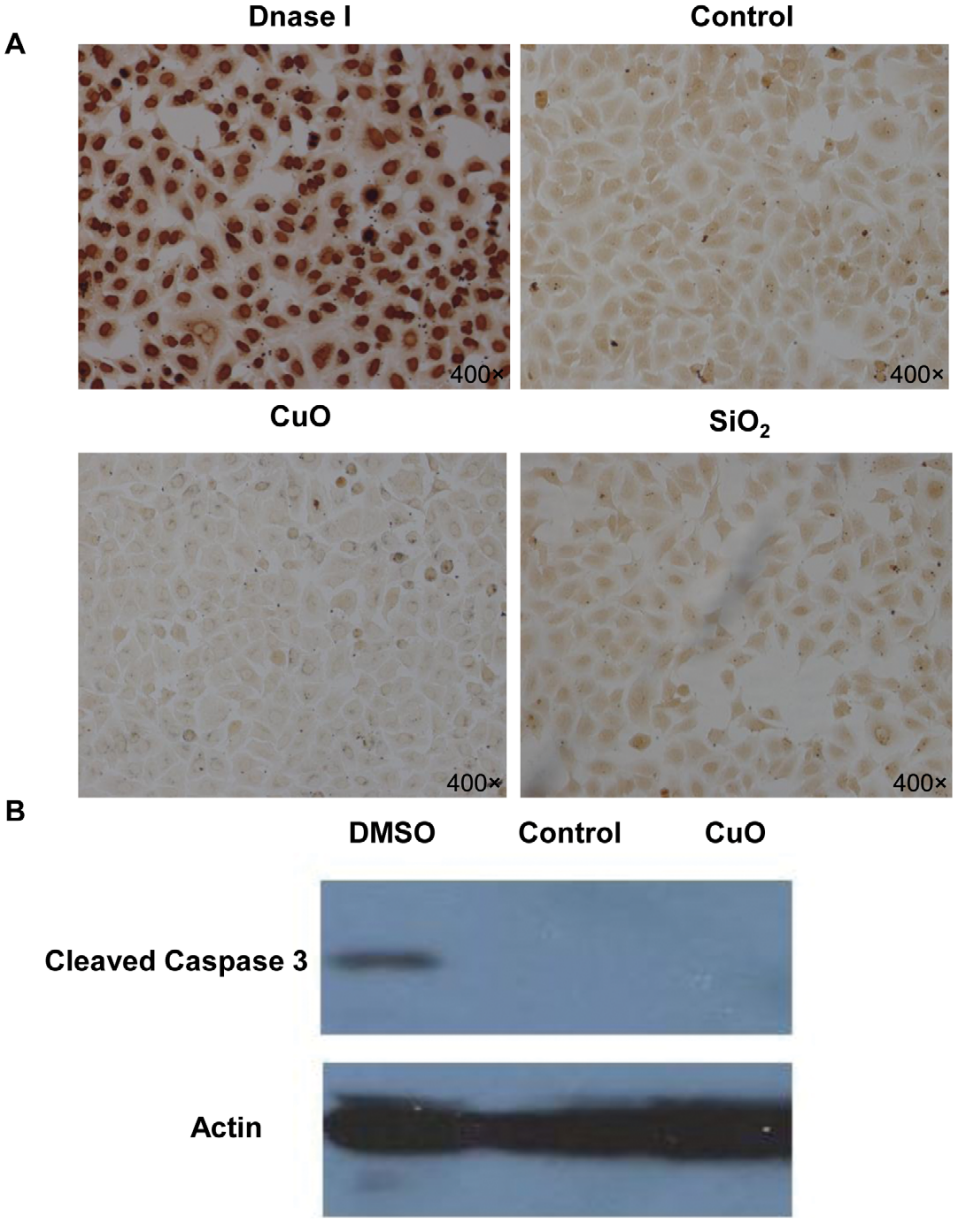
Copper oxide nanoparticles induce cell death through a non-apoptotic pathway in A549 cells. (A) TUNEL assay results for A549 cells treated with CuO or SiO_2_ NPs (30 µg/ml) for 8 h. The magnitude = 400×(B) Western blot analysis of cleaved caspase 3 in A549 cells treated with 30 µg/ml CuO NPs for 12 h. 4% DMSO was used as a positive control.

The cytotoxicity of the metal oxide nanoparticles were assessed using the MTS assay. MTS, (3(4,5-dimethylthiazol-2-yl)-5-(3-carboxymethoxyphenyl)-2- (4-sulfophenyl)-2H-tetrazolium), as an analog of MTT, can give a water-soluble formazan product in the presence of phenazinemethosulfate (PMS) and be used in the microculture screening assay for cell growth. The scale of Y-axis is the ratio of sample colored products absorbance at 490 nm vs. the control colored products absorbance at 490 nm. Therefore, the scale of Y-axis is proportional to the cell viability [15]. After exposure to nanoparticles for 24 h, the A549 cells exposed to copper oxide NPs had the lowest percentage of viable cells at the concentration of 30 µl/ml (25%, *p*<0.001) (Fig. 1A) and the cell viability was decreased in a dose-dependent manner (Fig. 1B). However, a slight decrease in cell viability was observed for cells treated with SiO2 NPs (89%, *p*<0.01), and all the other nanoparticles tested appeared to have no cytotoxic effect. Next, we evaluated the cytotoxicity of these nanoparticles in two other cell lines, NCI-H1650 and CNE-2Z, and found similar results (Fig. 1C, Fig. 1D). In summary, CuO NPs appeared to have a high cytotoxicity in all three respiratory cell types, while the other metal oxide nanoparticles appeared to have little or no cytotoxic effect. We used the TUNEL (terminal deoxynucleotidyl transferased UTP nick-end labeling) assay, which is a reliable method for detecting and quantifying apoptotic cell death, to determine whether the low cell viability induced by CuO nanoparticles was a result of apoptosis. As shown in Figure 2A, we did not detect a significant difference in the percentage of apoptotic cells between the control group and the nanoparticle-treated test groups. Furthermore, we analyzed the protein levels of caspase 3, which is key mediator of apoptosis in mammalian cells [16], and its cleavage is commonly used as an indicator of apoptotic cell death. We used 4% DMSO as a positive control and found no significant increase in the level of activated caspase-3 in CuO NPs-treated cells (Fig. 2B). Even though apoptosis is the most common form of eukaryotic cell death, our results do not support that CuO nanoparticles induced A549 cell death through the apoptotic pathway.

### Copper Oxide Nanoparticles Induce Autophagic Cell Death in A549 Cells

**Figure 3:**
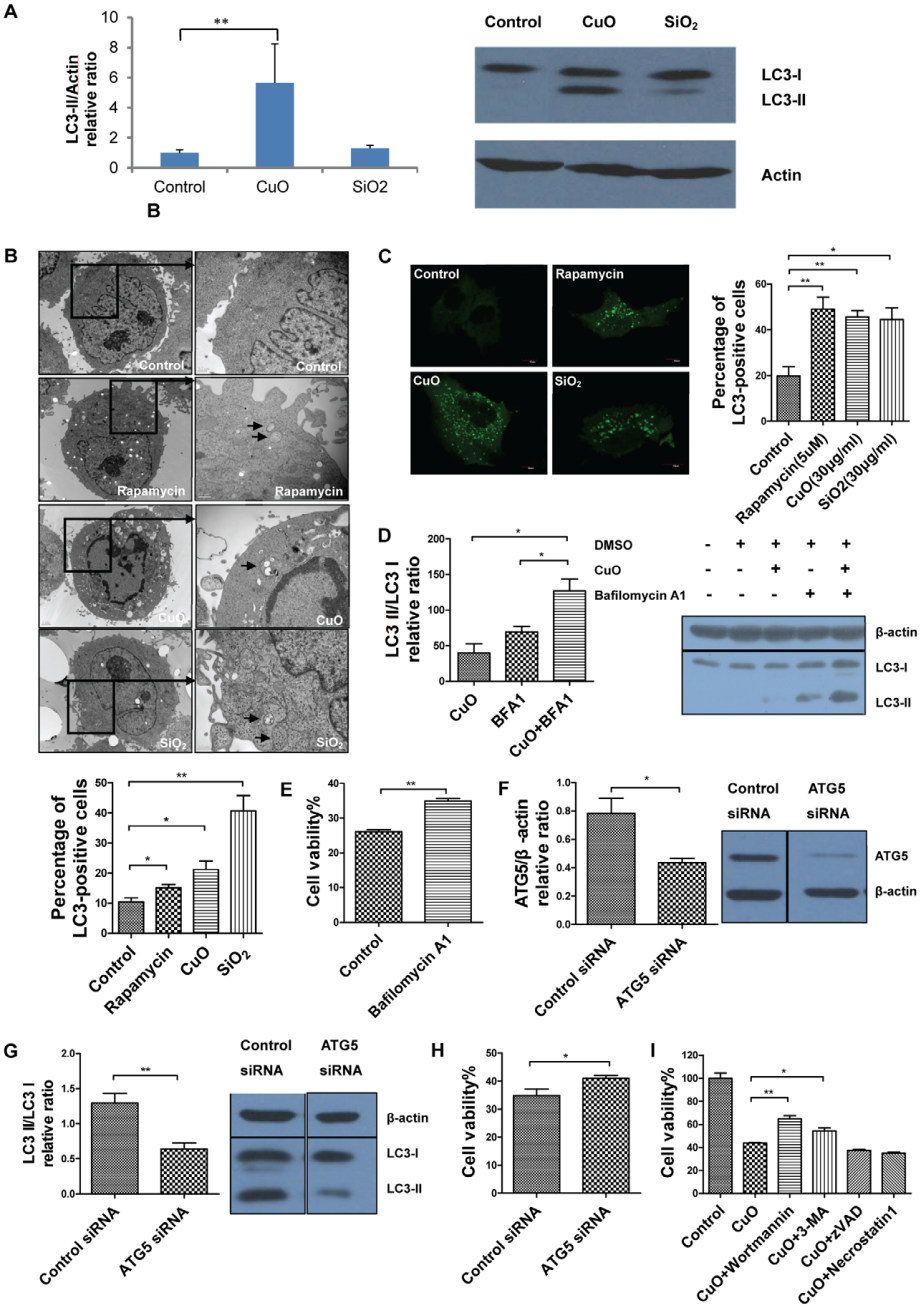
Copper oxide nanoparticles induce autophagy in A549 cells. (A) Western blot analysis of LC3-I and LC3-II in A549 cells treated with CuO or SiO2 NPs (30 µg/ml) for 12 h, bar graph indicates the LC3 II/β-actin relative ratio (B) EM images of A549 cells treated with control, rapamycin (5 µM), CuO (30 µg/ml) or SiO_2_ (30 µg/ml); bar graph indicates the percentage of autophagy-positive cells (C) Confocal images of A549 cells transfected with LC3-EGFP plasmid and treated with control, rapamycin (5 µM), CuO (30 µg/ml) or SiO_2_ (30 µg/ml); bar graph indicates the percentage of LC3-positive cells (D) Western blot analysis of LC3-I and LC3-II in A549 cells treated with bafilomycin A1 (50 nm) 1 h before exposure to CuO NPs (30 µg/ml) for 3 h, bar graph indicates the LC3 II/LC3 I relative ratio (E) MTS assay results for A549 cells treated with bafilomycin A1 (50 nm) 1 h before exposure to CuO NPs (30 µg/ml) for 18 h (F) Western blotting analysis of the efficiency of ATG5-knockdown efficiency by ATG5 siRNA, bar graph indicates the Atg5/β-actin relative ratio (G) Western blotting analysis of the expression of LC3-I and LC3-II in ATG5 siRNA treated cells or control siRNA treated cells, bar graph indicates LC3 II/LC3 I relative ratio (H) Cell viability of A549 cells upon treatment of CuO NPs (30 µg/ml) for 24 h in ATG5 siRNA treated group and control siRNA treated group (I) MTS assay results for A549 cells treated with 30 µg/ml CuO NPs and cell death inhibitors for 24 h; **p<0.01 versus control and *p<0.05 versus control.

Cells can degrade proteins and organelles by engulfing them in double-membrane vacuoles known as autophagosomes, this process is called autophagy [17]. Recently, autophagy has been considered to be a novel type of cell death which is independently of the apoptotic pathway [18]. Various nanoparticleshave been proved to induce autophagy [19]–[21]. In order to determine whether CuO NPs can also induce autophagy, we analyzed the protein levels of microtubule-associated protein 1 light chain 3 (LC3) using Western blot analysis. We detected a significant increase in LC3-II in CuO-treated cell lysates, as shown in Figure 3A. LC3-II is an important protein marker for autophagic activity [16], and this result suggests that autophagy may play a crucial role in CuO-induced cell death. Furthermore, TEM (Transmission electron microscopy) results showed that CuO NPs induced the accumulation of autophagosomes in A549 cells, which is a hallmark of autophagy (Fig. 3B). We also observed that in A549 cells, CuO NPs efficiently induced the formation of LC3 puncta (Fig. 3C), which is another alternative hallmark of autophagy [22]. Furthermore, to better interpret changes in level of processed LC3 II and to determine whether CuO NPs could induce the autophagy flux, we treated A549 cells with bafilomycin A1, which is an autophagy inhibitor and can prevent degradation of autolysosome content through inhibiting the Na+H+ pump at the lysosome [23], and we found out that CuO induced increased level of LC3-II formation was further increased significantly by the addition of bafilomycin A1 (Fig. 3D). The cell viability of bafilomycin A1 treated cells was significantly higher compared to the control group (Fig. 3E). Taken together, these results show that CuO NPs can indeed induce autophagy in A549 cells.

Although autophagy can cause cell death, it is also thought to be a survival mechanism for cells under certain conditions [18], [24]. To determine whether autophagy is involved in CuO NP-induced cell death, we used siRNA to knock down Atg5 in A549 cells (Fig. 3F) since the Atg5-Atg12 complex is a key regulator of autophagy [25]. We found that with the same CuO exposure, the level of LC3-II protein in cell lysates was substantially decreased upon treatment with siRNA against Atg5 compared to that in cells treated with control siRNA (Fig. 3G) and accordingly, the cell viability of Atg5 siRNA-treated group was significantly higher compared to the control siRNA-treated group (Fig. 3H). Furthermore, we exposed A549 cells to three types of autophagy inhibitors, 3-methyladenine (3 mM), wortmannin (1 µM) and bafilomycin A1 (50 nM), and found that the cell viability of CuO NP-treated A549 cells significantly increased (Fig. 3I, Fig. 3E). While zVAD-fmk (20 µM) and necrostatin-1 (20 µM), which are inhibitors of apoptosis and necrosis, did not appear to have any rescue effect on cell death (Fig. 3I). All the above results suggest that CuO NP-induced cell death in A549 cells was mediated, at least partially, through autophagy.

## Discussion

Copper oxide nanoparticles are widely used in many products, including antimicrobial agents, heat transfer fluids, semiconductors and healthcare products, such as intrauterine contraceptive devices [12], [26]. CuO particles are also major sources of pollutants in ambient air. Data on the impact of these airborne pollutants on the respiratory system, however, is limited. In this study, we evaluated the potential toxicity of several types of commonly used metal oxide NPs in 3 different cell lines. We found that CuO NPs caused significant cell death, while iron oxides, titanium dioxide and silica NPs appeared to have little or no cytotoxic effect. These results are consistent with the results obtained by two other groups, who also showed that different metal oxide NPs have highly variable cytotoxic effects in A549 and Hep-2 cells.

CuO NPs appeared to have greater cytotoxicity compared to their bulk counterpart and to other metal oxide NPs [11], [27]. The mechanism of CuO-NP-induced cytotoxcity, however, remains unknown. Based on the results of this study, we propose that autophagy may be involved in CuO NP- induced cytotoxicity. Autophagy, or cellular self-digestion, is a conserved mechanism involved in the degradation of proteins and organelles in the cytoplasm [18]. Even though autophagy primarily serves as a pro-survival mechanism in contexts including nutrient and growth factor deprivation, ER stress and microbial infection, it has been shown that prolonged and uncontrolled autophagy is involved in cell death [28]–[30]. Recently, some nanoparticle types have been identified as a novel class of autophagy activators and inducers of autophagic cell death [19], [21], [31], [32]. Our results suggest that CuO NPs can also induce cell death through the autophagic pathway. Some studies suggest that the toxic effect of CuO NPs may be caused by oxidative stress and the ability of CuO to damage the mitochondria [27], [33]. In a number of studies, it has been shown that oxidative stress can induce autophagy under specific circumstances, such as starvation or ischemic conditions [34]–[36]. However, it currently remains unclear whether or how oxidative stress is linked to the autophagy pathway in the context of CuO NP-induced cell death.

Our study shows that copper oxide nanoparticles have a higher cytotoxic potential compared to the other metal oxide NPs used in this study, and that this cytotoxicity may involve the autophagic pathway. We also found in subsequent experiments that CuO NPs are also acutely toxic in mice, and cause significant pulmonary edema and death within 24 h. It has also been shown that copper NPs have acute toxicological effects in models such as the zebrafish and rat [10], [37]–[39]. Therefore, it is possible that CuO NPs could pose a risk to human health after frequent exposure, and necessary precautions may need to be taken when handling these NPs. Furthermore, CuO NPs may serve as a reliable positive control in establishing bioassay models that assess the pulmonary toxicity of ultrafine particles.
